# Elevated CO_2_ improves both lipid accumulation and growth rate in the glucose-6-phosphate dehydrogenase engineered *Phaeodactylum tricornutum*

**DOI:** 10.1186/s12934-019-1214-x

**Published:** 2019-09-23

**Authors:** Songcui Wu, Wenhui Gu, Aiyou Huang, Yuanxiang Li, Manoj Kumar, Phaik Eem Lim, Li Huan, Shan Gao, Guangce Wang

**Affiliations:** 1 Key Laboratory of Experimental Marine Biology, Institute of Oceanology, Chinese Academy of Sciences, Qingdao, 266071 People’s Republic of China; 2 Laboratory for Marine Biology and Biotechnology, Qingdao National Laboratory for Marine Science and Technology, Qingdao, 266071 People’s Republic of China; 3 Center for Ocean Mega-Science, Chinese Academy of Sciences, 7 Nanhai Road, Qingdao, 266071 People’s Republic of China; 4 Climate Change Cluster, Faculty of Science, University of Technology Sydney (UTS), Sydney, NSW Australia; 5 Institute of Ocean and Earth Sciences (IOES), University of Malaya, 50603 Kuala Lumpur, Malaysia

**Keywords:** Glucose-6-phosphate dehydrogenase, Overexpression, Antisense knockdown, CO_2_, *Phaeodactylum tricornutum*, Lipid accumulation, Algal growth rate

## Abstract

**Background:**

Numerous studies have shown that stress induction and genetic engineering can effectively increase lipid accumulation, but lead to a decrease of growth in the majority of microalgae. We previously found that elevated CO_2_ concentration increased lipid productivity as well as growth in *Phaeodactylum tricornutum*, along with an enhancement of the oxidative pentose phosphate pathway (OPPP) activity. The purpose of this work directed toward the verification of the critical role of glucose-6-phosphate dehydrogenase (G6PDH), the rate-limiting enzyme in the OPPP, in lipid accumulation in *P. tricornutum* and its simultaneous rapid growth rate under high-CO_2_ (0.15%) cultivation.

**Results:**

In this study, G6PDH was identified as a target for algal strain improvement, wherein G6PDH gene was successfully overexpressed and antisense knockdown in *P. tricornutum*, and systematic comparisons of the photosynthesis performance, algal growth, lipid content, fatty acid profiles, NADPH production, G6PDH activity and transcriptional abundance were performed. The results showed that, due to the enhanced G6PDH activity, transcriptional abundance and NAPDH production, overexpression of G6PDH accompanied by high-CO_2_ cultivation resulted in a much higher of both lipid content and growth in *P. tricornutum*, while knockdown of G6PDH greatly decreased algal growth as well as lipid accumulation. In addition, the total proportions of saturated and unsaturated fatty acid, especially the polyunsaturated fatty acid eicosapentaenoic acid (EPA; C20:5, n-3), were highly increased in high-CO_2_ cultivated G6PDH overexpressed strains.

**Conclusions:**

The successful of overexpression and antisense knockdown of G6PDH well demonstrated the positive influence of G6PDH on algal growth and lipid accumulation in *P. tricornutum*. The improvement of algal growth, lipid content as well as polyunsaturated fatty acids in high-CO_2_ cultivated G6PDH overexpressed *P. tricornutum* suggested this G6PDH overexpression-high CO_2_ cultivation pattern provides an efficient and economical route for algal strain improvement to develop algal-based biodiesel production.

## Background

The marine diatom *Phaeodactylum tricornutum* is well-known for its high photosynthesis efficiency, rapid growth rate, and abundant lipids (especially unsaturated fatty acids), fucoxanthin and protein yield. These excellent properties make it not only promisingly for industrial microalgae production, but also one of the most widely studied model diatoms in terms of ecology, physiology, biochemistry and molecular biology [1, 2]. In recent years, increasing attention has been paid to research on *P. tricornutum* lipid production as this diatom can trap light energy and assimilate CO_2_ in the form of lipids and approximately 20% to 30% of its biomass is composed of triacylglyceride under different growth conditions [1, 3, 4]. *P. tricornutum* is therefore considered a suitable yet underexploited target for biofuel production.

To enhance lipid productivity in *P. tricornutum*, numerous studies have been carried out including oleaginous microalgal species screening and microalgal domesticated [5–11]. However, most of these studies showed that *P. tricornutum* tends to accumulate lipids under stress conditions, and this stress-induced lipid accumulation is usually accompanied by growth limitation [6–8]. With the exception of optimizing cultivation conditions, many of the efforts aimed at increasing microalgal lipid production have been made via molecular genetic techniques, such as gene overexpression and knockdown [12–15]. At present, the commonly used gene knockdown strategies based on RNA interference methods are antisense or inverted repeat approaches [16]. Antisense knockdown, namely, stable transformation of constructs expressing a gene or gene fragment in the antisense orientation, has been used to silence a target gene for a long time [17]. Comparing to inverted repeat, antisense knockdown is much simpler and easier to perform. In the recent years, many engineering attempts have been made in *P. tricornutum* to improve lipid production. For instance, overexpression of malic enzyme [14] and glycerol-3-phosphate dehydrogenase [18] and knockdown of pyruvate dehydrogenase kinase [13] and phosphoenolpyruvate carboxykinase [15] to increase lipid production. However, genetic engineering has been reported that could result in decreased algal growth in *P. tricornutum* to different extents although it can increase algal lipid content [12–14]. Therefore, exploring for an improvement of both lipid accumulation and growth rate is of great importance in microalgae production.

Our recent study found that elevated CO_2_ concentration increased lipid productivity as well as growth rate in *P. tricornutum* [9]. In addition, an increase of enzyme activity and mRNA expression level of glucose-6-phosphate dehydrogenase (G6PDH) and 6-phosphogluconate dehydrogenase (6PGDH), involved in the oxidative pentose phosphate pathway (OPPP), were observed in high-CO_2_ cultivated *P. tricornutum* [9]. It is known that both algal growth and lipid synthesis require a large amount of carbon skeleton, energy and reductants, in particular a large amount of NADPH is required for lipids synthesis. While the steps catalyzed by G6PDH and 6PGDH in the OPPP are the major source of NADPH and play an important role in providing reductant to meet cellular needs for reductive biosynthesis [19, 20], this suggests that elevated activity of the OPPP might account for enhanced *P. tricornutum* growth and lipid accumulation under high-CO_2_ cultivation. This provides a direction for genetic transformation of *P. tricornutum*. Metabolic engineering of the OPPP in *P. tricornutum* may verify this hypothesis for increasing lipid synthesis and biomass yield alga strains.

Actually, increasing attention has been focused on genetic engineering of the OPPP to increase lipid or fatty acid synthesis by enhancing the levels of intracellular NADPH in several species, such as *Aurantiochytrium* sp. SD116 [21] and *Fistulifera solaris* [22]. Both studies showed that overexpression of G6PDH resulted in an increase in either polyunsaturated fatty acid synthesis or lipid accumulation by enhancing NADPH generated from the OPPP. Such enhancement was also observed in *P. tricornutum* transformants with G6PDH overexpression according to Xue et al. [23], which demonstrated our hypothesis that elevated G6PDH activity and abundance might account for higher intracellular lipid accumulation. However, in their study, G6PDH overexpressed *P. tricornutum* was cultured with no air or CO_2_ enrichment, and enhanced G6PDH activity and transcript abundance did not lead to an increase in algal growth rate. This is different from our previous findings that enhanced G6PDH activity and transcriptional abundance, might also lead to an increase in algal growth under high-CO_2_ cultivation. A question therefore remains about why G6PDH overexpression did not improve algal growth in *P. tricornutum*. Is it possible that insufficient carbon in the cultures limited its growth?

It is known that, increasing CO_2_ concentration enhances the efficiency of photosynthetic carbon fixation and the growth of phytoplankton [24]. Thus, in the present study, to further investigated the potential role of G6PDH, the key rate-limiting enzyme in the OPPP, in algal growth and lipid synthesis under high-CO_2_ cultivation, G6PDH antisense knockdown in *P. tricornutum* was firstly carried out. In addition, in order to improve algal biomass and lipid accumulation, transgenic *P. tricornutum* strains with overexpression of G6PDH were constructed. As expected, overexpression of G6PDH accompanied by high-CO_2_ cultivation enhanced both lipid content and growth of *P. tricornutum*, while knockdown of G6PDH greatly decreased algal growth as well as lipid accumulation. These results not only demonstrated the positive influence of G6PDH on lipid accumulation and growth in *P. tricornutum*, but also provided an efficient and economical route for algal strain improvement to increase microalgal biomass and lipid yields in industrial biodiesel production.

## Results

### Construction and screening of transgenic *P. tricornutum*

**Fig. 1:**
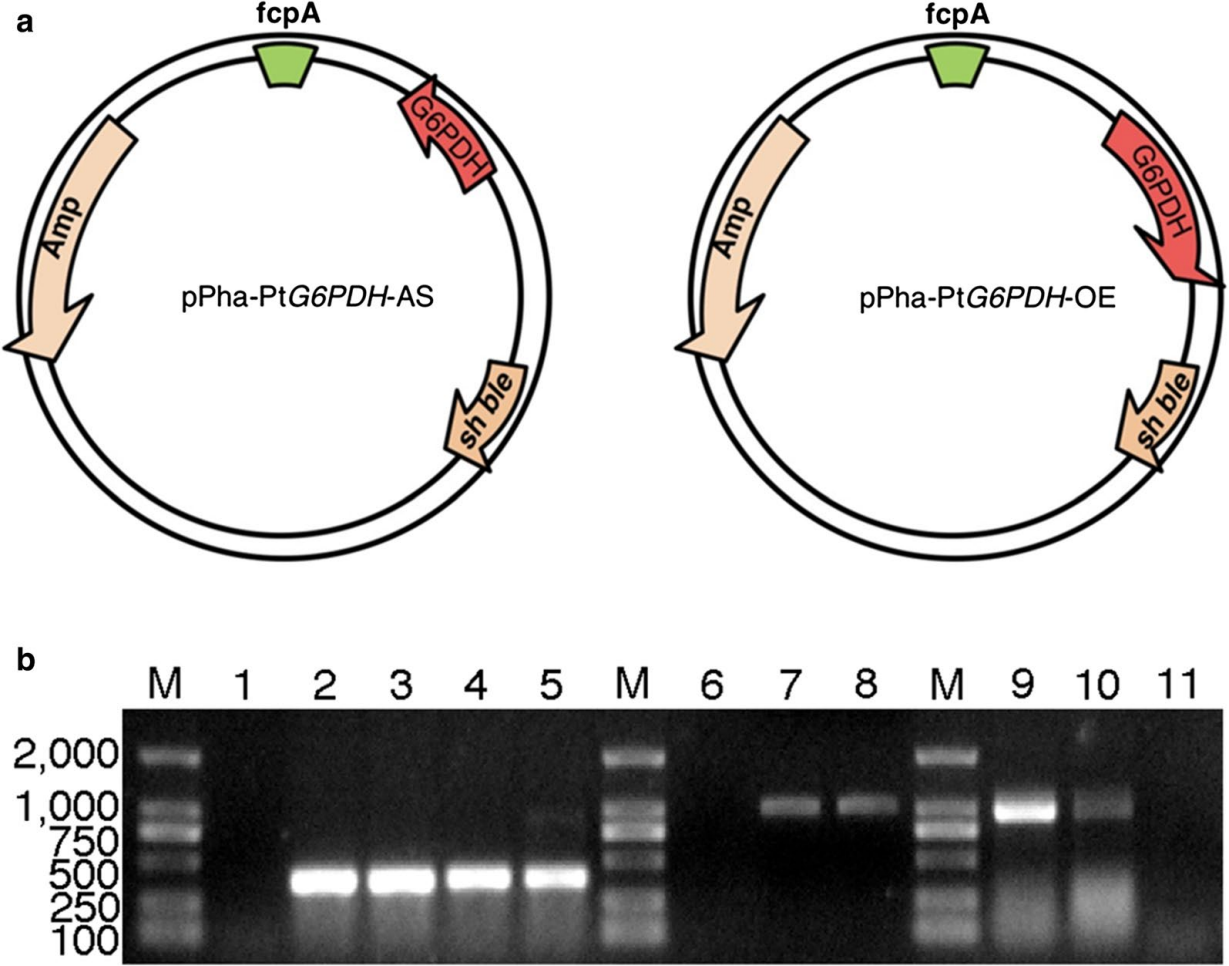
Vectors for G6PDH overexpression and antisense knockdown in *P. tricornutum*. **a** Schematics of pPha-Pt*G6PDH*-OE and pPha-Pt*G6PDH*-AS vectors for overexpression or silencing of *P. tricornutum* G6PDH. The cDNA (amplified using *G6Poe_fw* and *G6Poe_rv*) were digested with *Eco*RI and *Hin*dIII and subsequently ligated in sense orientation into the *Eco*RI–*Hin*dIII sites of pPha-T1, which are located downstream of the *fcp*A promoter, resulting in the final transformation vectors pPha-Pt*G6PDH*-OE; the amplicons (amplified using *G6Pas_fw* and *G6Pas_rv*) were digested with *Eco*RI and *Hin*dIII and then ligated in antisense orientation of pPha-T1 and finally resulted in the vector pPha-Pt*G6PDH*-AS. **b** Preliminary molecular analysis of transgenic *P. tricornutum* strains. Line 1 to line 5, PCR analysis of the resistant gene *sh ble* in the wild-type, PtG6PDH-OE1, PtG6PDH-OE2, PtG6PDH-S1 and PtG6PDH-S2 with primers *ble*_*fw* and *ble*_*rv*, respectively. Line 6 to line 8, PCR analysis in the wild-type, PtG6PDH-OE1 and PtG6PDH-OE2 with primers OE_*fw*1 and OE_*rv*1. Line 9 to line 11, PCR analysis in the PtG6PDH-S1, PtG6PDH-S2 and the wild-type strain using primers AS_*fw*1 and AS_*rv*1. M, 2-kb DNA size marker

In order to study the important role of G6PDH, we attempted to silence and overexpress the G6PDH gene in *P. tricornutum*. Two constructs, pPha-Pt*G6PDH*-OE and pPha-Pt*G6PDH*-AS, were generated based on the pPha-T1 vector. The overexpression construct contained the full-length Pt*G6PDH* coding sequence, and the antisense construct contained a 180-bp fragment complementary to the partial sequence of G6PDH mRNA. The two different constructs were introduced into *P. tricornutum* via particle gun bombardment and firstly screened on a solid medium using Zeocin. To verify the integration of the selectable marker *sh ble* gene, PCR with *sh ble* gene-specific primers was performed. Eight G6PDH overexpressed and seven silenced colonies were identified as successfully amplified expected size fragment of *sh ble* gene. For clarity of presentation, four engineered strains (PtG6PDH-OE1 and PtG6PDH-OE2 as G6PDH overexpressed lines; PtG6PDH-S1 and PtG6PDH-S2 as G6PDH knockdown lines) were randomly selected for further examination and metabolic analysis. As shown in Fig. 1b, the 374-bp *sh ble* gene band was expectedly present in the transgenic lines and absent in the wild-type strain, indicated the exogenous plasmid pPha-T1 containing *sh ble* gene was transferred into *P. tricornutum*. Moreover, to further confirm the stable integration of the expression construct, PCRs with primers corresponding to the regions flanking the Pt*G6PDH* and *sh ble* gene were performed. As expected, a 0.95-kb band was detected in PtG6PDH-S1 and PtG6PDH-S2 lines, and a 1.11-kb band was observed in PtG6PDH-OE1 and PtG6PDH-OE2 lines (Fig. 1b), respectively. Both fragments were not amplified from wild-type cells (Fig. 1b). These results suggested the exogenous Pt*G6PDH* gene successfully integrated into the genome of these four engineered lines.

### Transcript abundance and activity of G6PDH varied when cultured with different CO_2_ concentrations

**Fig. 2:**
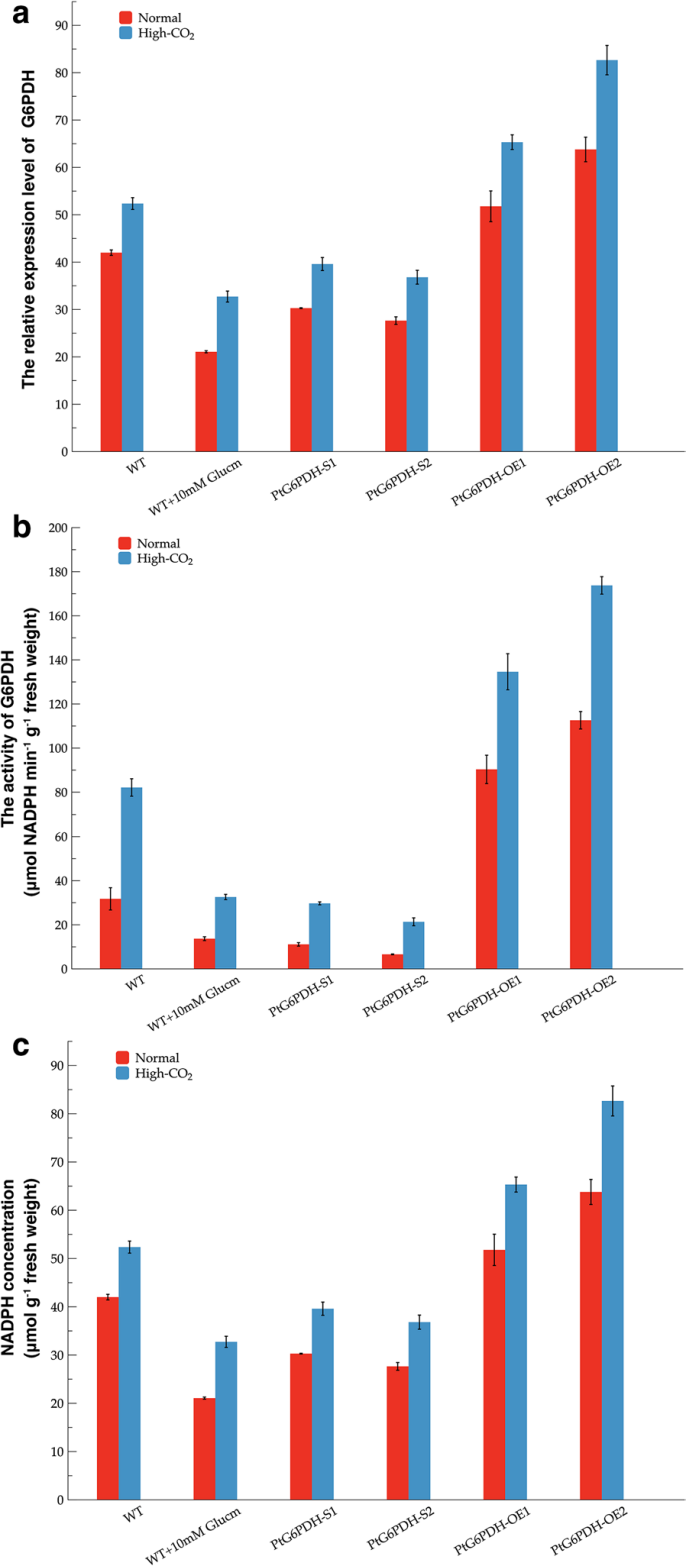
G6PDH transcriptional abundance, activity and NAPDH production in the CO_2_ cultivated microalgae strains. **a** The transcriptional abundance of G6PDH in the wild-type (with or without glucosamine treatment) and four G6PDH engineered strains under CO_2_ cultivation. **b** Differences in the activity of G6PDH between the wild-type strain and G6PDH engineered strains. **c** The NADPH production in different algal strains cultured with different concentrations of CO_2_. Data were shown as mean values ± SD for three independent experiments. *WT* the wild-type *P. tricornutum* strain, *Glucm* glucosamine

To verify the potential role of G6PDH in lipid accumulation and algal growth, the transcript abundance and activity of G6PDH in the four transgenic strains were determined. As shown in Fig. 2a, the transcript abundance of G6PDH markedly increased in the two overexpressed lines by over 4.04-fold and 7.74-fold under normal cultivation, and by sixfold and 14.60-fold under high-CO_2_ cultivation, respectively, compared with wild-type cells. In two silenced strains, the transcript abundance of G6PDH significantly decreased by 21.94% to 48.24% with normal CO_2_ cultivation, and by around 43.50% and 47.68% under high-CO_2_ cultivation, respectively (*P* < 0.01). These results demonstrated that the exogenous *G6PDH* gene was highly expressed in G6PDH overexpressed transgenic *P. tricornutum*, but down-regulated in G6PDH silenced lines.

The activity of G6PDH in the four transgenic *P. tricornutum* and wild-type strains were monitored spectrophotometrically by constantly measuring the reduction of NADP^+^ at 340 nm. As shown in Fig. 2b, G6PDH activity significantly differed between the wild-type and engineered strains. In both overexpressed lines, G6PDH activity markedly increased by approximately 2.84-fold and 3.54-fold under normal cultivation and by 1.64-fold and 2.12-fold under high-CO_2_ cultivation. In the two silenced strains, the G6PDH activity significantly decreased by 65.00% and 79.27% under normal cultivation and by 60.77% and 62.02% under high-CO_2_ cultivation respectively (*P* < 0.01), compared to wide type cells. These results demonstrated that overexpression of the *G6PDH* gene stimulated an enhancement of enzyme activity as well as transcript abundance, while silencing of G6PDH led to a decrease in G6PDH activity and relative expression. In all four transformants, transcript abundance showed was highly correlated with enzyme activity, suggesting the successful overexpression and suppression of G6PDH in the engineered strains. Moreover, the G6PDH activity and transcript abundance corresponded to algal growth and lipid content, except for PtG6PDH-OE1, in which enhanced G6PDH activity and transcript level increased lipid accumulation but not growth.

### Differences in photosynthetic performance between wild-type and transgenic strains

As shown in Table 1, YII and ETRII values showed that inhibitor 3-(3′,4′-dichlorophenyl)-1,1-dimethylurea (DCMU) which is known to block electron transport after the primary acceptor in photosystem II (PSII) [25], led to a significant decrease in PSII activity, which finally resulted in a decline of photosystem I (PSI) activity. When treated with 10 μM DCMU, the G6PDH overexpression strain PtG6PDH-OE2 had the lowest decrease in all parameters values, followed by PtG6PDH-OE1, and then the wild-type strain, while the two silenced lines showed the largest decline, especially PtG6PDH-S2. These results suggested that in G6PDH overexpressed strains the inhibition of DCMU is markedly lower than that of wild-type and silenced strains, which may be due to the increase in the amount of NADPH, generated by overexpression of G6PDH, reduces the inhibition of photosynthesis electron transport during DCMU treatment. The different photosynthetic performance in these strains confirmed the successful overexpression and silencing of G6PDH in *P. tricornutum*.

**Table 1:** Photosynthesis performance of the WT and four transgenic strains with or without DCMU treatment

	Y(I)		ETR(I)		F_v_/F_m_		Y(II)		ETR(II)	
	Control	10 μM DCMU	Control	10 μM DCMU	Control	10 μM DCMU	Control	10 μM DCMU	Control	10 μM DCMU
Wild	0.90 ± 0.01	0.28 ± 0.01	32.70 ± 0.30	10.13 ± 0.25	0.66 ± 0.00	0.30 ± 0.01	0.56 ± 0.00	0.02 ± 0.00	20.37 ± 0.06	0.77 ± 0.06
PtG6PDH-S1	0.87 ± 0.01	*0.23 ± 0.01*	31.40 ± 0.28	8.50 ± 0.46	0.61 ± 0.01	*0.25 ± 0.00*	*0.55 ± 0.00*	0.02 ± 0.00	*19.70 ± 0.10*	0.63 ± 0.06
PtG6PDH-S2	*0.85 ± 0.00*	*0.19 ± 0.01*	*30.50 ± 0.14*	*6.70 ± 0.57*	*0.57 ± 0.01*	*0.22 ± 0.01*	*0.53 ± 0.00*	*0.01 ± 0.00*	*19.03 ± 0.31*	*0.50 ± 0.00*
PtG6PDH-OE1	*0.94 ± 0.01*	*0.41 ± 0.01*	*34.03 ± 0.25*	*14.70 ± 0.20*	*0.70 ± 0.01*	*0.46 ± 0.04*	0.58 ± 0.00	*0.03 ± 0.00*	20.97 ± 0.21	*1.10 ± 0.10*
PtG6PDH-OE2	*0.98 ± 0.01*	*0.45 ± 0.01*	*35.63 ± 0.06*	*16.23 ± 0.67*	*0.74 ± 0.01*	*0.51 ± 0.04*	*0.61 ± 0.00*	*0.04 ± 0.00*	*22.13 ± 0.12*	*1.40 ± 0.10*

Data was shown as mean values ± SD for three independent experiments. Italic indicates a significant difference with WT controls (ANOVA and MNOVA, *P* < 0.05)

### Changes in algal growth and chlorophyll concentration

**Fig. 3:**
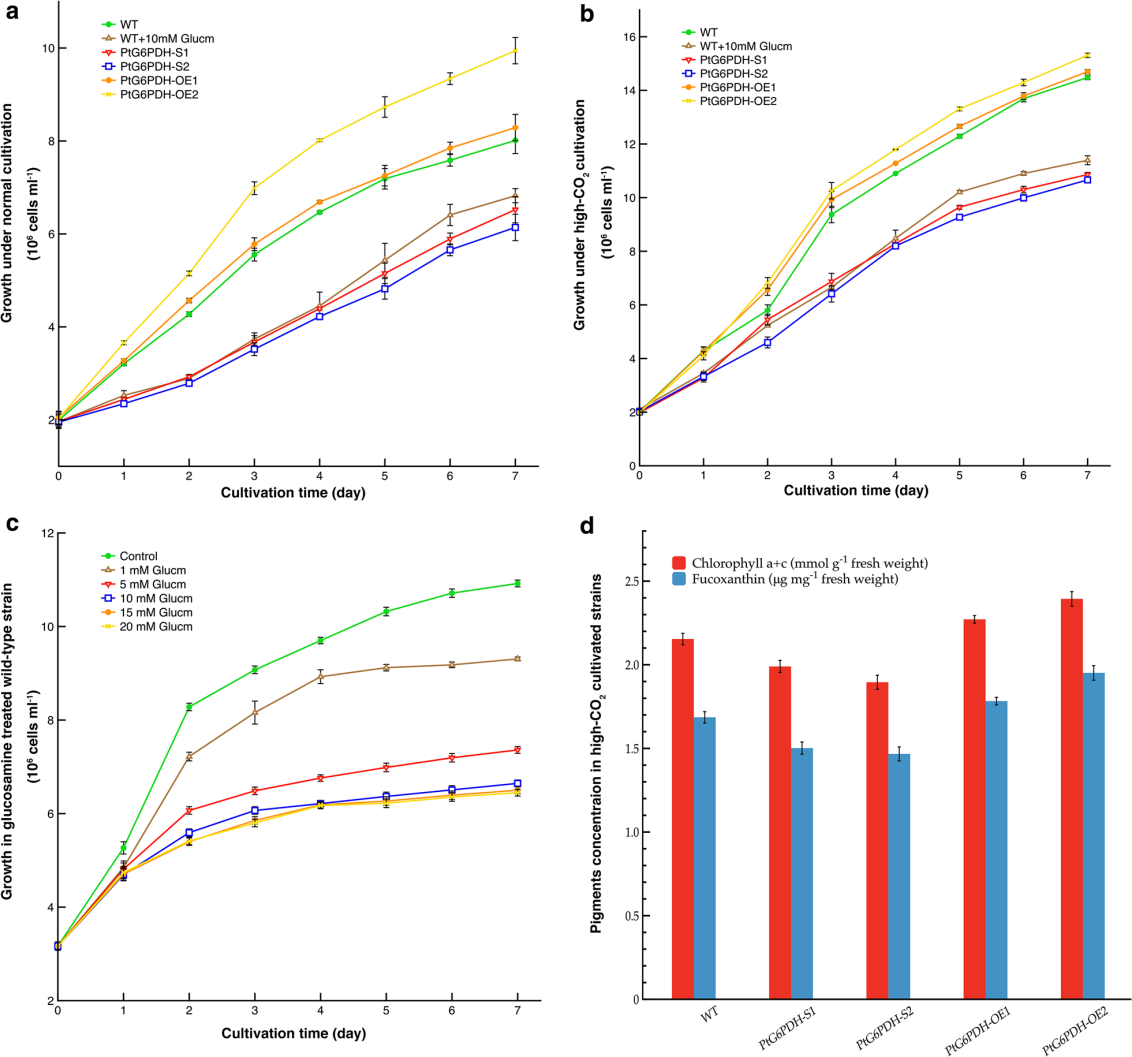
Changes of growth and pigments concentration in microalgae strains. **a** Growth in the wild-type (with or without glucosamine treated) and the four transformants under normal cultivation. **b** Growth in high-CO_2_ cultivated microalgal strains. **c** Growth in wild-type *P. tricornutum* strains with different concentrations of glucosamine treatment. **d** Changes of chlorophyll *a* + *c* and focuxanthin content in high-CO_2_ cultured wild-type and transgenic strains. Data were shown as mean values ± SD for three independent experiments. *WT* the wild-type *P. tricornutum* strain, *Glucm* glucosamine

To evaluate the impact of G6PDH suppression or overexpression on algal cell characteristics, cell growth and lipid content in the engineered strains cultured with 0.035% and 0.15% CO_2_ were determined, respectively. As shown in Fig. 3, during the exponential growth phase (from day 0 to day 7), the PtG6PDH-OE2 exhibited the highest growth rate, followed by the wild-type and PtG6PDH-OE1, in which no obvious differences in growth rate were observed, and the two G6PDH silenced lines had the lowest growth rate. These growth tendencies were observed following both normal (Fig. 3a) and high-CO_2_ (Fig. 3b) cultivation. These findings indicated that during CO_2_ cultivation, overexpression of G6PDH slightly accelerated *P. tricornutum* growth, while G6PDH knockdown led to a marked decline in algal growth. Moreover, compared to normal cultivation, all algal strains showed significant growth enhancement under high-CO_2_ concentration, which was consistent with our previous study.

In diatoms, chlorophyll *a* + *c*, fucoxanthin and cofactor proteins usually form the fucoxanthin-chlorophyll *a*/*c* protein complex, which mainly performs as a light harvesting pigment complex that is similar to LHCII in higher plants and green algae [26, 27]. In order to determine the influence of G6PDH overexpression or knockdown on cellular general characteristics, chlorophyll *a* + *c* and fucoxanthin concentrations in high-CO_2_ cultivated wild-type and four transgenic strains were determined. As shown in Fig. 3d, compared with the wild-type cells, the PtG6PDH-OE2 had the highest chlorophyll *a* + *c* and fucoxanthin contents, followed by the PtG6PDH-OE1, and then wild-type strains, while the two G6PDH silenced strains exhibited an obvious decrease in chlorophyll *a* + *c* and fucoxanthin concentrations (*P *< 0.05). No significant difference in chlorophyll *a* + *c* and fucoxanthin concentration was found between the two silenced strains (*P* > 0.05). Photosynthetic carbon fixation efficiency is positively correlated with chlorophyll content under autotrophic cultivation, which may therefore be associated with algal growth. As expected, changes in chlorophyll *a* + *c* and fucoxanthin contents were found to essentially match algal growth (Fig. 3b, d).

### Lipid accumulation and fatty acid composition differed between wild-type and transgenic strains

**Fig. 4:**
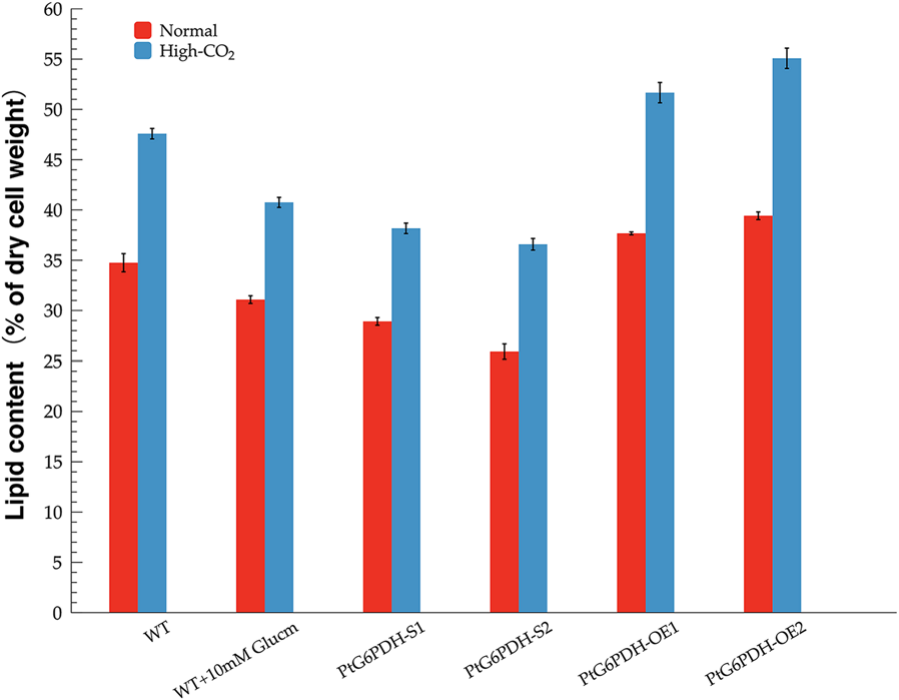
The lipid content in the wild-type (with or without glucosamine treatment) and four G6PDH engineered strains under CO_2_ cultivation. *WT* the wild-type *P. tricornutum* strain, *Glucm* glucosamine

The lipid content in CO_2_ cultured wild-type and transformant strains were shown in Fig. 4. In the presence of both concentrations of CO_2_, the highest lipid content was found in the PtG6PDH-OE2, followed by PtG6PDH-OE1, and then wild-type cells, while the two G6PDH knockdown strains had the lowest lipid content. As expected, in addition to enhanced algal growth, the lipid content in all types of algal cells increased under high-CO_2_ cultivation (Fig. 4). In both G6PDH overexpressed strains, the lipid content was significantly increased (*P* < 0.01), especially PtG6PDH-OE2 increased by 15.76%, while the lipid content in PtG6PDH-S1 and PtG6PDH-S2 was decreased by approximately 19.79% to 23.12% (*P* < 0.01), compared with the wild-type strain, under high-CO_2_ cultivation. The G6PDH overexpressed lines showed enhanced lipid accumulation of around 38% compared with the G6PDH silencing strains. These results suggested that overexpression of G6PDH stimulated *P. tricornutum* lipid accumulation, while knockdown of G6PDH resulted in a significant decrease in lipid content under CO_2_ cultivation.

**Fig. 5:**
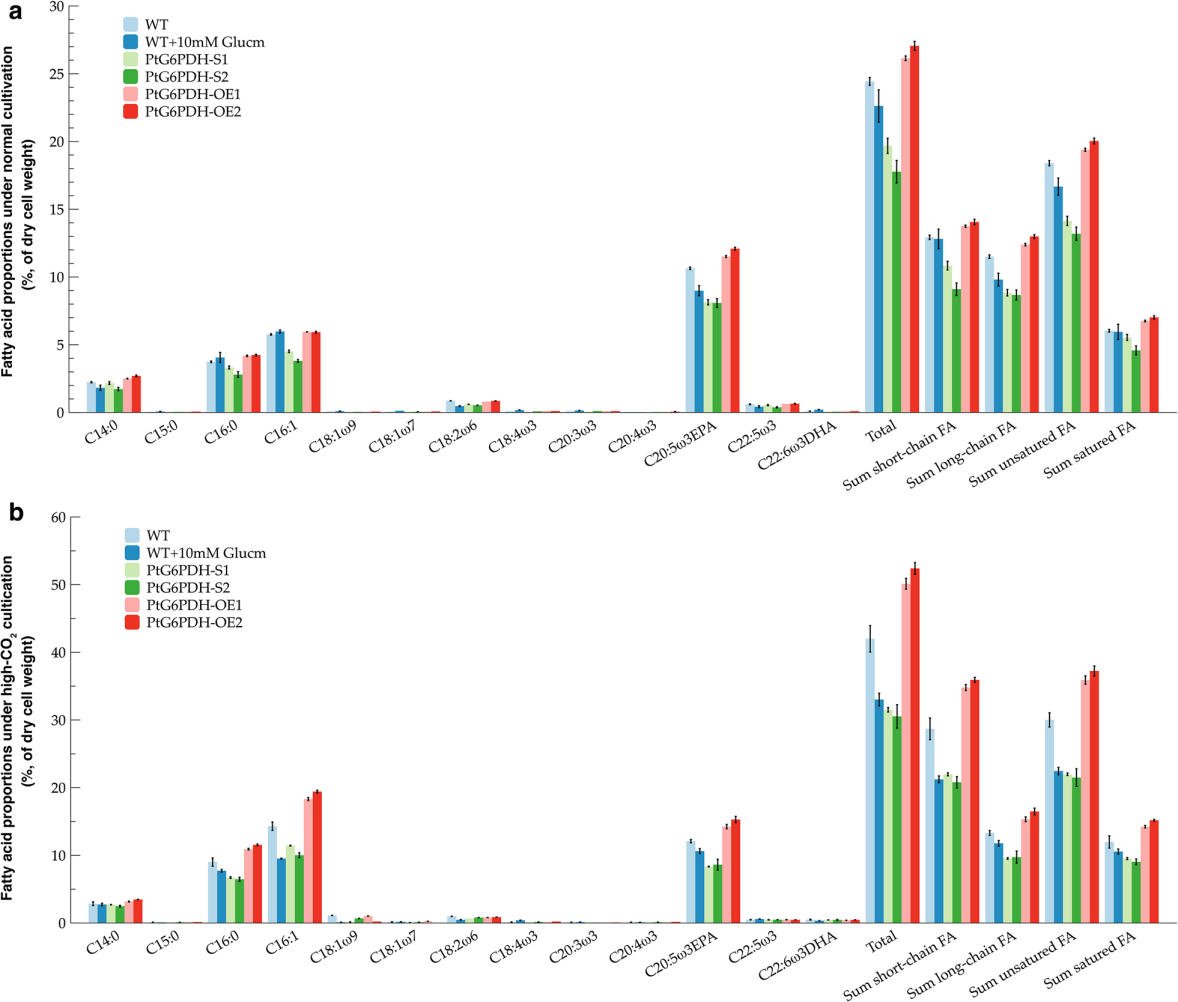
Analysis of fatty acid profiles in different concentrations of CO_2_ cultured *P. tricornutum* strains. **a** The proportions of fatty acid profiles in microalgae under normal cultivation. **b** The compositions of fatty acid in high-CO_2_ cultivated algal strains. Data were shown as mean values ± SD for three independent experiments. *WT* the wild-type *P. tricornutum* strain, *Glucm* glucosamine, *FA* fatty acid

As lipid content changed in transgenic *P. tricornutum* strains, investigation of the effects of G6PDH engineering on the fatty acid profiles was performed. As shown in Fig. 5a, b, the fatty acid composition of total lipids was similar in the wild-type and four transgenic strains, and the main fatty acids involved in these algal strains included C14:0, C16:0, C16:1, and C20:5. The total percentages of short-chain fatty acids were slightly increased in the G6PDH overexpressed strains under normal cultivation (*P* < 0.05), but increased by approximately 2.5-fold in the overexpressed strains which were higher than that in the wild-type strain by 21.31% and 25.22% under high-CO_2_ cultivation (*P *< 0.05), compared with the wild-type strain, respectively. The total proportions of long-chain fatty acids in PtG6PDH-OE1 and PtG6PDH-OE2 were slightly higher than that in the wild-type strain under normal cultivation (*P *< 0.01), respectively. Under high-CO_2_ cultivation, the long-chain fatty acids in the overexpressed strains increased by 23.70% and 26.78% (*P *< 0.05), and was higher than that in wild-type cells by 15.21% and 23.84% (*P *< 0.05). In the two G6PDH knockdown lines, the total proportions of short-chain fatty acids were significantly decreased by 16.15% and 29.58% under normal cultivation (*P *< 0.01), and decreased by 23.43% and 27.05% when cultured with high level of CO_2_ (*P *< 0.01), compared with wild-type cells, respectively. The total long-chain fatty acid proportions in the two silenced strains decreased by 23.12% and 24.64% under normal cultivation, and by 28.31% and 26.31% respectively under high-CO_2_ cultivation (*P* < 0.05), compared with wild-type. In addition to the enhancement of total short- and long-chain fatty acid in all algal strains, significant increases in both total saturated and unsaturated fatty acid proportions were also found in the high-CO_2_ cultured algal strains. PtG6PDH-OE1 and PtG6PDH-OE2 showed higher proportions of total saturated and unsaturated fatty acids than wild-type cells under CO_2_ cultivation, especially unsaturated fatty acids which increased by 19.60% and 24.04% (*P* < 0.01), respectively. The total percentage of unsaturated fatty acids in the silenced strains increased under high-CO_2_ cultivation, but it was reduced by 26.77% and 27.93% compared with the wild-type strain (*P* < 0.01), respectively. These results showed that overexpressed G6PDH with high-CO_2_ cultivation could markedly accelerate the biosynthesis of short-chain, long-chain, and unsaturated fatty acids in *P. tricornutum*, while silencing of G6PDH resulted in a decrease in fatty acids synthesis.

### G6PDH engineering resulted in different NADPH concentrations

As shown in Fig. 2c, compared with wild-type cells, the NADPH concentration in PtG6PDH-OE1 and PtG6PDH-OE2 was greatly enhanced by 23.31% and 51.89% under normal cultivation, and by 24.75% and 57.84% under high-CO_2_ cultivation, while in PtG6PDH-S1 and PtG6PDH-S2 the NADPH content was markedly reduced by 27.89.06% and 34.17% under normal cultivation, and by 24.38% and 29.70% under high-CO_2_ cultivation (*P* < 0.01). These results suggested that G6PDH overexpression stimulated a significant increase in NADPH generation, and knockdown of G6PDH led to a decrease in NADPH concentration. In the two G6PDH silenced lines, decreased NADPH concentration may account for the observed decline in growth and lipid accumulation. The increase in NADPH in the two G6PDH overexpressed strains, along with the increase in lipid content as well as algal growth, indicated that excess NADPH was used for lipid accumulation and growth in *P. tricornutum* under CO_2_ cultivation.

### Changes in nitrate concentration and intracellular carbon to nitrogen ratio (C/N ratio)

**Fig. 6:**
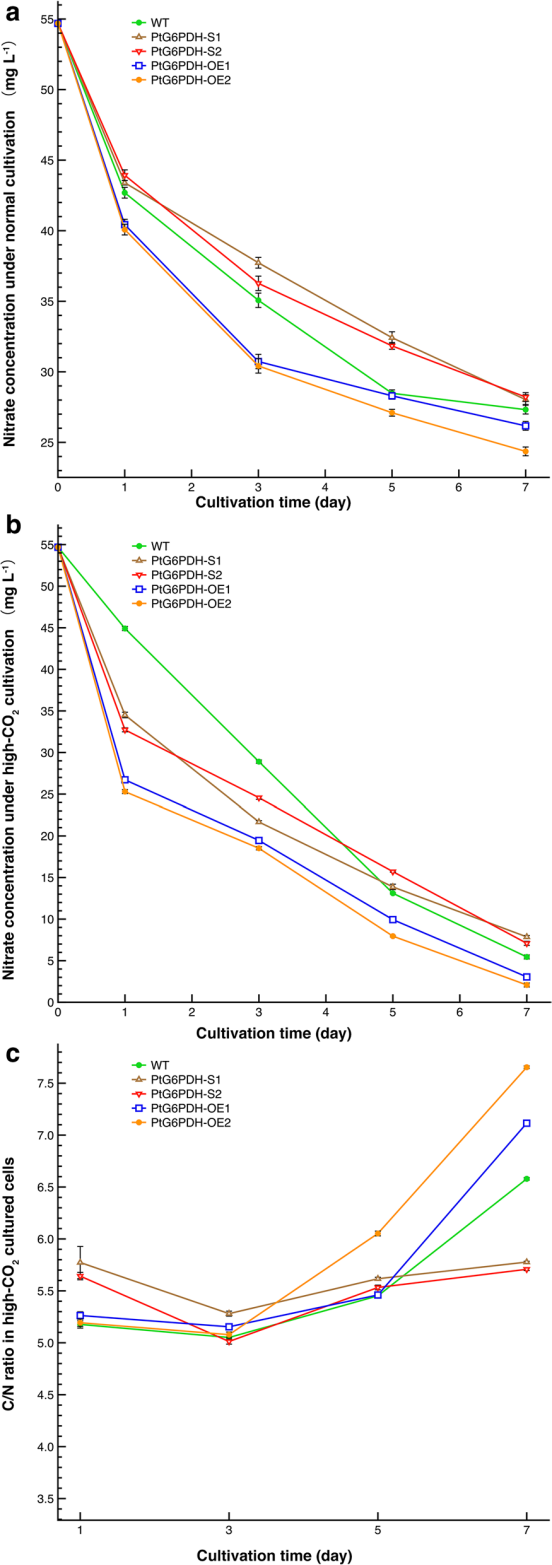
Changes of the nitrate concentration, intracellular nitrogen and carbon content in the wild-type and transgenic strains during aerated cultivation. **a** Nitrate concentrations in the normal cultivated algal cultures. **b** Nitrate concentrations in the high-CO_2_ cultivated algal cultures. **c** Differences in intracellular C/N ratio between high-CO_2_ cultivated wild-type and G6PDH engineered strains. Data were shown as mean values ± SD for three independent experiments. *WT* the wild-type *P. tricornutum* strain

As lipid accumulation in algal cells usually begins at nitrogen limitation, accompanied by an increase in the intracellular C/N ratio, the nitrate concentration in each of the batch cultures was determined to further analyze the mechanism of higher growth and lipid content in high-CO_2_ cultivated *P. tricornutum*. As shown in Fig. 6a, b, during the 7 day cultivation, the nitrate concentrations in each of the batch cultures tended to continuously decline, but both the wild-type and engineered strains showed higher nitrate utilization under high-CO_2_ cultivation than under normal cultivation. In addition, whether cultured with high-CO_2_ or normal cultivation, the two G6PDH overexpressed algal cultures showed the lowest final nitrate concentration, indicated that PtG6PDH-OE1 and PtG6PDH-OE2 utilized nitrate most efficiently. The final concentrations in the two G6PDH knockdown cultures were the highest, which suggested that nitrate utilization in PtG6PDH-S1 and PtG6PDH-S2 was the slowest. These changes well matched with the algal growth of each algal strain.

The changes in intracellular C/N ratio from day 0 to day 7 were shown in Fig. 6c. The four transformants and wild-type strains all showed a decline in C/N ratio from day 1 to day 3, which then increased from day 3 to day 7 under high-CO_2_ cultivation. The final C/N ratio on day 7 was the highest in PtG6PDH-OE1 and PtG6PDH-OE2 (Fig. 6c), along with the lowest final nitrate concentration (Fig. 6b). The C/N ratios in the two G6PDH knockdown lines declined and then increased slightly and the final C/N ratios in these strains were lower than those in the G6PDH overexpressed lines and the wild-type strain. Differences in the final C/N ratio matched the observed changes in final lipid contents.

### Confirmation of the potential role of G6PDH in algal growth and lipid synthesis by inhibitor treatment

Plant cells can not only spontaneously synthesize glucosamine (Glucm), an amino-sugar, by amidation of fructose-6-phosphate, but can also take up exogenous Glucm which is rapidly phosphorylated to glucosamine 6-phosphate, a well-known competitive inhibitor of G6PDH [28–30]. Glucm is usually used as a G6PDH inhibitor. To verify the results obtained from the G6PDH silenced lines, Glucm was used to inhibit G6PDH activity. The lowest effective dose of inhibitor was screened based on algal growth rate. As shown in Fig. 3c, the growth rate of *P. tricornutum* gradually declined with increasing Glucm concentration, and algal growth under Glucm treatment were significantly lower than that in the control. However, no significant decrease in the growth of *P. tricornutum* was observed when the Glucm concentration ranged from 10 to 20 mM. This suggested that the minimum Glucm concentration, which markedly affected algal growth, was 10 mM.

During aerated cultivation, 10 mM Glucm was added to wild-type cultures, and whether under normal or high-CO_2_ cultivation, the growth rate of Glucm treated wild-type cells was slightly higher than that of the two G6PDH silenced strains, but were significantly slower than wild-type cells without inhibitor (Fig. 3a, b). In addition, as shown in Fig. 4, compared with wild-type cells without inhibitor treatment, the addition of Glucm resulted in lower lipid content in *P. tricornutum* which decreased by 10.55% under normal cultivation and by 14.36% under high-CO_2_ cultivation (*P* < 0.05), respectively. Following aeration and inhibitor treatment, lipid content in *P. tricornutum* was slightly increased compared with PtG6PDH-S1 and PtG6PDH-S2 (*P* < 0.05). When cultured with high-CO_2_, the proportions of short-chain and unsaturated fatty acid were significantly different between Glucm treated and G6PDH knockdown strains, and were markedly decreased by 25.99% and 25.20% compared with no inhibitor treated wild-type cells (*P* < 0.01), respectively. No significant difference was observed in long-chain fatty acid proportion between the Glucm treated wild-type and silenced stains (*P* > 0.05), but decreased by 11.44% compared with wild-type strain without inhibitor treatment (*P* < 0.05).

Besides a lower growth rate and lipid content, decreased transcriptional abundance of G6PDH was also observed in inhibitor treated wild-type cells (Fig. 2a), which decreased by 23.77% under normal cultivation and by 41.74% under high-CO_2_ condition (*P* < 0.05), respectively. The G6PDH activity was reduced by 56.90% under normal cultivation and by 60.30% under high-CO_2_ condition (*P* < 0.05), compared with wild-type strain without inhibitor added, respectively. The G6PDH activity and transcriptional abundance were either not significantly different or slightly higher than the two G6PDH knockdown lines (Fig. 2a, b). In addition, following Glucm treatment, the wild-type cells showed a significant decrease in NADPH concentration of approximately 49.83% under normal cultivation and 37.49% under high-CO_2_ cultivation (*P* < 0.01), compared to wild-type cells without inhibitor treatment (Fig. 2c). These results suggested that the addition of Glucm, similar to silencing G6PDH, markedly reduced the transcriptional abundance of G6PDH and inhibited its activity consequently led to the decrease in algal growth, lipid accumulation and fatty acids synthesis irrespective of whether the algal cells were cultured with normal or high-CO_2_ cultivation.

## Discussion

### The rate-limiting enzyme G6PDH involved in the OPPP played a critical role in *P. tricornutum* growth

Following genetically engineering of G6PDH silenced and overexpressed strains were successfully constructed in this study, and the growth rates in transgenic *P. tricornutum* strains were differed from the wild-type strain. It is known that the OPPP is a major resource of NADPH, and also the major provider of pentose such as ribose 5-phosphate and ribulose 5-phosphate (Ru5P) [31, 32]. Ru5P can generate ribulose 1, 5-diphosphate (RuBP), the substrate for Rubisco during the CO_2_ fixation step in the Calvin cycle, catalyzed by ribulose 5-phosphate kinase (PRK). Elevated G6PDH activity, transcript abundance and NADPH production in overexpressed strains indicated enhancement of the OPPP, which suggested an increase in the amount of Ru5P generated in this pathway. Our previous studies have shown that when wild-type *P. tricornutum* cells were cultured with a high level of CO_2_, the activities and transcriptional abundance of Calvin cycle-related enzymes including PRK were upregulated [9], indicating that more Ru5P in the overexpressed algal strains could generate more RuBP than the wild-type strain under high-CO_2_ cultivation. As Rubisco is the most abundance protein in plant cells [33], when the substrate RuBP is sufficient, the concentration of CO_2_ may be the essential factor in affecting the carbon fixation efficiency of the Calvin cycle.

As shown in Fig. 3a, b, enhancement of algal growth was observed in *P. tricornutum* strains, including G6PDH engineered and inhibitor treated strains, when cultured with high-CO_2_, which indicated the effects of the carbon source on algal growth. In the present study, the growth rate in G6PDH overexpressed strain PtG6PDH-OE2 was higher than that in the wild-type strain, while in the study of Xue et al. [23], no significant increase in algal growth was observed in the no air or CO_2_ pumped G6PDH overexpressed strains. Thus, we suggested that might have been caused by an insufficient carbon sources, while a sufficient carbon source under high-CO_2_ cultivation promoted the growth rate of G6PDH overexpressed strain in our study. In addition, whether under normal or high-CO_2_ cultivation, G6PDH knockdown and inhibitor treatment significantly decreased the growth rate of *P. tricornutum* to different extents, indicated that G6PDH played a critical role in algal growth, and the negative effects on algal growth due to the addition of inhibitor and by silencing G6PDH were consistent.

### Overexpression of G6PDH stimulated lipid accumulation in *P. tricornutum* while G6PDH knockdown decreased

It is known that nitrogen deprivation typically stimulates lipid accumulation as it can lead to an increase in the C/N ratio in algal cells and eventually causes an increase in lipid accumulation. In other words, higher C/N ratio could generally cause higher lipid accumulation in algal cells. As shown in Fig. 6a, b, the final nitrite concentrations in high-CO_2_ cultures were significantly lower than that in the normal cultivated cultures. Fig. 6c showed that the final C/N ratios in both wild-type and transformants strains under high-CO_2_ cultivation were increased significantly. These demonstrated that a combination of high level of CO_2_ and low level of nitrogen can increase the C/N ratio. This findings is consistent with the findings obtained by Li et al. [34], where the C/N ratio in *P. tricornutum* increased under high-CO_2_ conditions in both nitrogen limited and nitrogen replete conditions. Additionally, although the final C/N ratio increased in all strains, the highest final C/N ratio was found in the G6PDH overexpressed strains and well matched their higher final lipid content, while the lowest final C/N ratio and lowest lipid content were observed in the G6PDH knockdown strains. Moreover, the lipid content in the overexpressed G6PDH strains was significantly higher than that in the wild-type and silenced algal strains, whether under normal or high-CO_2_ cultivation. An increase in lipid accumulation was also found in another oleaginous diatom *Fistulifera solaris* due to the enhancement of NADPH production from the OPPP according to Osada et al. [22]. These findings suggested that a combination of G6PDH engineering, CO_2_ concentration and nitrogen concentration affected algal intracellular C/N ratio, which resulted in differences in lipid content. This G6PDH overexpression-high-CO_2_ cultivation pattern provides a new route for improvements in both growth rate and lipid accumulation in microalgae production.

### G6PDH highly related to fatty acid synthesis in *P. tricornutum* and is a promising target gene for fatty acids profiles improvement

As shown in Fig. 5a, b, under both normal and high-CO_2_ conditions, total proportions of fatty acids enhanced in the two G6PDH overexpressed strains, while decreased in the G6PDH knockdown strains, compared to the wild-type strain. Such increase in fatty acid biosynthesis was also found in other microorganisms such as G6PDH overexpressed *Yarrowia lipolytica*, due to the enhancement of NADPH generated from the OPPP [35]. In addition, overexpression of G6PDH can elevate intracellular polyunsaturated fatty acid (PUFA) accumulation in algal cells [21]. In the present study, the proportions of both unsaturated and saturated fatty acids (SFA) were significantly increased in the two G6PDH overexpressed strains. In the high-CO_2_ cultivated wild-type and silenced strains, unsaturated fatty acids and SFA enhanced in different degrees though these were not as significant as in the overexpressed strains. These results suggested that G6PDH was highly related to fatty acid synthesis. As we know, unsaturated fatty acid especially the PUFAs such as eicosapentaenoic acid (EPA; C20:5, *n*-3) and docosahexaenoic acid (DHA; 22:6, *n*-3), are of increasing interest due to their positive effects on human physical and mental health [36, 37]. PUFA can be synthesized by a number of different routes based on variations in elongase and desaturase biochemistry by modifying the SFA precursors [38–40]. Therefore, PUFA synthesis is an energy demanding process [40, 41]. NADPH is an essential reductant for the synthesis of either SFA or PUFA [42, 43], and the availability of NADPH can increase the reaction velocity of NADPH-requiring enzymes involved in fatty acid synthesis such as acetyl-CoA carboxylase [41]. In other words, fatty acid synthesis is controlled by the availability of NADPH. The first step catalyzed by G6PDH in the OPPP is one of the major intracellular NADPH supplying for the synthesis of fatty acids. In this study, overexpression of G6PDH led to an enhancement of G6PDH activity which was corresponding to higher NADPH production, which well matched with the higher fatty acids proportion and PUFAs proportion in the two overexpressed strains. Thus, engineered NADPH regenerating systems such as genetic engineering of G6PDH in the OPPP is a promising way to increase the productivity of lipids and improve fatty acid profiles.

Besides, it should be noted that with a high level of CO_2_ cultivation, the proportion of short-chain fatty acids was markedly increased, as well as the proportion of long-chain fatty acids, in PtG6PDH-OE1 and PtG6PDH-OE2 (Fig. 5b). It is known that a large amount of reductant such as NADPH is required in fatty acid synthesis, and in photosynthetic plants cells, de novo biosynthesis of short-chain fatty acids occurs in the chloroplast while fatty acids elongation occurs at the cytoplasm. Thus, in the present study, in addition to the elevated NAPDH requirement in the cytoplasm for fatty acids elongation, much more NADPH was required in chloroplasts for short-chain fatty acids synthesis in high-CO_2_ cultured G6PDH overexpressed strains. As a major provider of NADPH, G6PDH has been reported widely present in plant cytosol, plastids, and peroxisomes [44–46]. According to Xue et al. [23], G6PDH was predominantly localized in the chloroplast in *P. tricornutum* by immuno-EM determination. Thus, we suggested that the location of G6PDH may account for its specific functions, for example, accelerated short- and long-chain fatty acids synthesis in G6PDH overexpressed strains via NADPH overproduction. The results derived from the percentages of short- and long-chain fatty acids in silenced and Glucm treated strains were also demonstrated that G6PDH played an important role in both short- and long-chain fatty acids biosynthesis in *P. tricornutum*.

## Conclusions

In conclusion, the successful of overexpression and antisense knockdown of G6PDH, the rate limiting enzyme involved in OPPP, well demonstrated the critical role of G6PDH played in algal growth and lipid accumulation in *P. tricornutum*. In this study, overexpression of G6PDH coupled with high CO_2_ cultivation were found improvely enhanced algal growth, lipid content as well as short-, long-chain and unsaturated fatty acids in *P. tricornutum* while those in G6PDH knockdown strains were decreased, suggested this G6PDH overexpression-high CO_2_ cultivation pattern provides a new route for an improvement of both lipid accumulation and growth rate in microalgae production.

## Methods

### Strains and growth conditions

The axenic wild-type *Phaeodactylum tricornutum* strain (Institute of Hydrobiology, Chinese Academy of Sciences) and transformants were grown in sterile artificial seawater enriched with f/2 nutrients at 20 ± 1 °C under a constant light intensity of 100 μmol m^−2^ s^−1^ with a 14:10 h light–dark (L/D) cycle. During aerated cultivation, algal strains were cultivated in sterilized carbon source deprived artificial seawater enriched with f/2-Si medium and was pumped with filtrated 0.035% CO_2_ (normal) and 0.15% CO_2_ (high-CO_2_) in 3-L flasks containing 2.5 L medium, respectively. During inhibitor treatment, following the addition of glucosamine (Glucm), a G6PDH inhibitor, at concentrations ranging from 0 to 20 mM, the effective concentration of inhibitor was selected by growth determination. Following aerated cultivation, the selected dosage of inhibitor was added to algal cultures in which other carbon source was deprived described above. Algal growth was determined daily by evaluation of the optical density (OD) at 730 nm with a UV-1800 spectrophotometer (Shimazu, Japan) and cell numbers were counted using a hemocytometer. All cultures were inoculated into f/2 medium at a cell density of 0.10 at OD_730nm_, and the pH of cultures was adjusted to 8.0 as cultivation beginning. Each treatment included three replicates.

### Construction of overexpression and antisense knockdown vectors

All fragments used for overexpressing and silencing vectors construction were amplified by PCR from *G6PDH* gene (Pt*G6PDH*, GenBank: XM_002183678.1), and then inserted into Pha-T1 vector which has a bleomycin-resistant gene (*sh ble*) cassette [47]. To generate pPha-Pt*G6PDH*-OE (overexpression) plasmid, the full length cDNA of the open reading frame encoding G6PDH was amplified with specific primers *G6Poe*_*fw* (containing an *Eco*RI site) and *G6Poe_rv* (containing a *Hin*dIII site). Additionally, antisense RNA expression was employed to knockdown transcripts for G6PDH. To construct pPha-Pt*G6PDH*-AS (antisense) plasmid, a 180-bp reverse complementary fragment of Pt*G6PDH* (corresponding to the Pt*G6PDH* gene sequence from 169 to 348 bp) was amplified from *P. tricornutum* cDNA using the primers *G6Pas_fw* (containing a *Hin*dIII site) and *G6Pas_rv* (containing an *Eco*RI site) (Additional file 1: Table S1). Both amplified cDNA sequence were confirmed by sequencing analysis at both orientations. The amplicons were digested with *Eco*RI and *Hin*dIII and subsequently ligated in sense and antisense orientation into the *Eco*RI-*Hin*dIII sites of pPha-T1, which are located downstream of the *fcp*A promoter, resulting in the final transformation vectors pPha-Pt*G6PDH*-OE and pPha-Pt*G6PDH*-AS (Fig. 1a), respectively.

### Biolistic transformation

A total of 1 × 10^8^ *P. tricornutum* cells in exponential phase were collected and plated in the center of f/2-Si 1.0% agar plates. The pPha-Pt*G6PDH*-OE and pPha-Pt*G6PDH*-AS vectors were introduced into *P. tricornutum* respectively, by microparticle bombardment which was performed essentially using a Biolistic PDS-1000/He Particle Delivery System (Bio-Rad, CA, USA) following the protocol [47]. After incubation in low light (~ 30 μmol m^−2^ s^−1^) overnight, the bombarded cells were eluted with 0.2 mL f/2-Si medium and the suspensions were spread on f/2-Si solid medium supplemented with 100 μg mL^−1^ zeocin (Invitrogen, Carlsbad, CA, USA). Resistant colonies appeared after 2–3 weeks incubation in white light (100 μmol m^−2^ s^−1^; 12 h photoperiod) at 20 °C, and were selected and inoculated into liquid f/2 medium with 75 μg mL^−1^ zeocin.

### Molecular analysis of transformants

The transformants were screened by checking the integration of *sh ble* gene with primers *ble*_*fw* and *ble*_*rv* and genomic DNA extracted from transformants was used as the template. To further confirm the integration of the constructed gene expression cassettes into transformed *P. tricornutum* cells, PCRs were performed with primers OE_*fw*1 and OE_*rv*1 for checking exogenous pPha-Pt*G6PDH*-OE, while AS_*fw*1 and AS_*rv*1 for checking pPha-Pt*G6PDH*-AS (Additional file 1: Table S1). The former primers were designed using an 1111-bp sequence which involving a partial Pt*G6PDH* sequence, a 576-bp sequence that ligated Pt*G6PDH* and *sh ble* gene, and a partial *sh ble* gene sequence. The latter one was designed using a 946-bp sequence which including a partial Pt*G6PDH* antisense sequence, a 576-bp of the ligated sequence and a partial *sh ble* gene sequence. PCR products were confirmed by sequencing analysis at both orientations.

### RNA extraction and quantitative real time-PCR (qRT-PCR)

For RNA extraction, cells of both wild-type (with or without inhibitor) and transformants were harvested by centrifugation and quickly frozen in liquid N_2_ after aerated cultivation for 7 days. An RNAprep Pure Plant kit (Tiangen, China) was used for total RNA extraction. The reverse transcription reaction was performed for single strand cDNA synthesis using a PrimeScript RT regent Kit with the gDNA Eraser (TaKaRa Biotech Co., Dalian, China) following the user’s manual. qRT-PCR was performed as described by Wu et al. [9], and the relative expression levels of Pt*G6PDH* gene in the wild-type and transformants were quantified by the Bio-Rad iQ5 Multicolor Real-Time PCR Reaction system (Bio-Rad, Hercules, CA, USA) with reagents from the Fast Essential DNA Green Master (Roche, Germany). For G6PDH overexpressed lines, qRT-PCR was performed with primers OE_*fw*2 and OE_*rv*2. For silenced lines, qRT-PCR was performed with primers AS-*fw*2 and AS-*rv*2. The ribosomal protein small subunit 30S (RPS) was used as the internal control to normalize the expression levels with primers RPS_*fw* and RPS_*rv* (see Additional file 1: Table S1). All values are presented as the means of triplicate qPCRs for each sample (n = 3) with standard deviation (SD).

### Determination of G6PDH activity

The activity of G6PDH was determined following the protocol according to Wu et al. [9] with some modifications. The crude protein in the wild-type (with or without inhibitor) and transformants strains was extracted using the pre-chilled extraction buffer. The activity of G6PDH was determined spectrophotometrically using a UV-1800 spectrophotometer by measuring continuously at 340 nm with assay buffer in triplets. Both the extract and the assay buffer were prepared according to the manufacturer’s protocol of a commercial G6PDH activity detection kit (Comin Biotechnology Co., Ltd., Suzhou, China). Reactions were started by addition of the algal extracts. Results were expressed as μmol NADPH min^−1^ g^−1^ fresh weight.

### Determination of photosynthetic performance

G6PDH was reported plays an important role in algal cyclic electron transport via NADPH supplying [48, 49]. Two G6PDH overexpressed lines and two silenced lines were randomly selected for photosynthetic performance determination, to verify successful genetic engineering of G6PDH in *P. tricornutum*. The inhibitor DCMU, a PSII inhibitor, was used to investigate the effects of PSII and G6PDH on PSI activity. Algal cultures were collected on day 7 by centrifugation and were adjusted to 1.2 at OD_730nm_ for subsequent PSI and PSII activity detection. After each algal samples were incubated in darkness with 10 μM DCMU for 5 min, five parameters, the maximum quantum yield of PSII (*F*_*v*_/*F*_*m*_), the effective quantum yield (YI and YII) and the relative rate of photosynthetic electron transport (ETRI and ETRII), were monitored by a Dual-PAM-100 fluorometer (Heinz Walz, Effeltrich, Germany) connected to a PC equipped with WinControl soft-ware (Heinz Walz). The values of these parameters were calculated following the formulas as described by Lin et al. [50].

### Lipid extraction and fatty acid analysis

CO_2_ cultivated algal cells were harvested and freeze-dried after aerated cultivation for 7 days. Lipids were extracted from 40 mg dried algal powder using a chloroform–methanol protocol according to Wu et al. [9]. The extracts were combined after three extractions and then evaporated under high purity nitrogen. The gravimetric means was used for quantification of total lipids until extracts maintained at a constant weight. Lipids were methylated and then fatty acid methyl esters were analyzed by gas chromatography according to the protocol described by Liu et al. [51]. Quantification of fatty acids was carried out with an Agilent 7890A instrument equipped with a DB-FFAP capillary column (30 m × 0.25 mm). Fatty acids were identified by comparison of their retention times with those of standards (Sigma). Nonadecanoic acid (19:0) was used as an internal standard.

### Detection of NADPH concentration

As the first step of the OPPP is catalyzed by G6PDH and is a major source of NADPH, the NADPH concentration of all CO_2_-cultured algal cells was analyzed. Harvested algal cells were ground into fine powders in liquid N_2_, and then were extracted and analyze using a commercial NADP (H) concentration colorimetric quantitative detection kit (Comin Biotechnology Co., Ltd., Suzhou, China) according to manufacturer’s protocol. The NADPH centration was calculated by following equation.

$${\text{NADPH }}\left( {{\text{nmol}}\;{\text{g}}^{ - 1} \;{\text{fresh}}\;{\text{weight}}} \right)\, = \,\left[ {7.2\, \times \,\left( {{\text{A}}2 - {\text{A}}1 - 0.072} \right)\, \times \,{\text{V}}1} \right] \div \left( {{\text{W}}\, \times \,{\text{V}}1 \div {\text{V}}2} \right)$$

A1 is the absorbance of blank control; A2 is the absorbance after reaction; V1 is the volume of extract sample in the reaction system; V2 is the volume of extraction buffer; 7.2 is the slope of the linear equation; 0.072 is the y-axis intercept of the linear equation.

### Carbon and nitrogen concentration determination

CO_2_ cultivated cells were collected after 8 h illumination on day 1, 3, 5 and 7, by centrifugation at 4000×*g* for 3 min, and then freeze-dried after being re-suspended twice in fresh nitrogen-deprived f/2 media. Carbon and nitrogen contents were determined by combustion of dried algal cells in a Vario MICRO cube Elemental Analyzer (Elementar, Germany) with a CN analysis mode. Phenylalanine was used as the standard. The flow rate of O_2_ was supplied at 25 mL min^−1^ for 70 s and the temperature of primary combustion, secondary combustion and the reduction tube was controlled at 960 °C, 930 °C and 830 °C, respectively.

For determination of nitrate concentration, supernatants following algal cells harvesting were collected and filtrated through 0.45 μm syringe pre-sterilized and dried filters, respectively. The concentration of nitrate in the algal cultures (filtrated supernatants) under different concentration of CO_2_ was determined with a Multi N/C 2100S Analyzer (Analytikjena, Germany) equipped with a solid-state electrochemical detector (ChD). As nitrate was the only added nitrogen source, sodium nitrate was used as the standard. The injection volume was 250 μL and the linear range was 0.2–5 mg L^−1^ (correlation coefficient 0.9995). The temperature of the combustion tube was controlled at 800 °C, and the maximum integration time was 200 s.

### Chlorophyll estimation

For chlorophyll extraction, 20 mg CO_2_ cultivated wild-type and transgenic cells were ground in liquid N_2_ into a fine algal powder, and then 5 mL precooled acetone/methanol (1:1, v/v) were added to samples by three times. Chlorophylls, including fucoxanthin and chlorophyll *a* and *c*1 + *c*2, were extracted in darkness until the cell pellets turned white. The extracts were combined after three extractions and filtered through a 0.22 μm syringe filter. Detection of fucoxanthin concentration was performed by HPLC (Agilent 1200, Agilent Technologies, USA) following the protocols as described by Zhao et al. [52]. Chlorophyll *a* and *c*1 + *c*2 contents were determined by following the equations as described by Jeffrey and Humphrey [53]. The fluorescence emission of the extracts at 664 nm and 630 nm were obtained by a UV-1800 spectrophotometer.

### Statistical analysis

Analysis of variance (ANOVA) and multifactor analysis of variance (MANOVA) were used to identify significant differences between groups, and a *P* value < 0.05 was considered significant. Statistical analyses were performed using SPSS software. All data were derived from three experiments and shown as mean values ± SD.

## Supplementary information

**Additional file 1: Table S1.** List of primers used in this study.

## Data Availability

The datasets used and/or analyzed during the current study are available from the corresponding author on reasonable request.

## Abbreviations

G6PDH: glucose-6-phosphate dehydrogenase
OPPP: oxidative pentose phosphate pathway
WT: wild-type
FA: fatty acid
C/N: carbon to nitrogen ratio
Glucm: glucosamine
DCMU: 3-(3′,4′-dichlorophenyl)-1,1-dimethylurea
PSI: photosystem I
PSII: photosystem II
Y(I): the effective quantum yield of PSI
Y(II): the effective quantum yield of PSII
F_v_/F_m_: the maximum quantum yield of PSII
ETR(I): the relative rate of photosynthetic electron transport of PSI
ETR (II): the relative rate of photosynthetic electron transport of PSII
Ru5P: ribulose 5-phosphate
RuBP: ribulose 1,5-diphosphate
PRK: ribulose 5-phosphate kinase
SFA: saturated fatty acid
PUFA: polyunsaturated fatty acid
ANOVA: analysis of variance
MANOVA: multifactor analysis of variance
SD: standard deviation
