# Supplementary material for: Elevated CO2 improves both lipid accumulation and growth rate in the glucose-6-phosphate dehydrogenase engineered Phaeodactylum tricornutum
Source: Microb Cell Fact. 2019 Sep 23;18:161. doi: 10.1186/s12934-019-1214-x (PMC6757359; doi:10.1186/s12934-019-1214-x)
Supplement: Supplementary file 1 — Additional file 1: Table S1. List of primers used in this study. [file 12934_2019_1214_MOESM1_ESM.docx]

Table S1 List of primers used in this study. (Underlining in primer sequences indicates the digested nucleotide sequences of the restriction enzyme.)

| Primer name | Primer sequence (5’-3’) |
| --- | --- |
| *G6Poe_fw* | GAATTCATGATAATTT GCAGTCTCACT |
| *G6Poe_rv* | AAGCTTAAGTGCAGACGGAGGAGGTGA |
| *G6Pas_fw* | AAGCTTGCGAGAAATGGCACAAGG |
| *G6Pas_rv* | GAATTCGTTCAT CGCAGTCGGGAGA |
| *ble*_*fw* | TGGCCAAGTTGACCAGTGCC |
| *ble*_*rv* | TCAGTCCTGCTCCTCGGCCA |
| OE_*fw*1 | TCCGCATACTACTGCTCCT |
| OE_*rv*1 | CACGACCTCCGACCACT |
| AS_*fw*1 | TCCGCATACTACTGCTCCT |
| AS_*rv*1 | GACACGACCTCCGACCA |
| OE_*fw*2 | GCGAGAAATGGCACAAGG |
| OE_*rv*2 | GTTCATCGCAGTCGGGAGA |
| AS-*fw*2 | TTGACAACGGAGCAGAAG |
| AS-*rv*2 | TGGCACAAGGTAAATGGA |
| RPS_*fw* | CGAAGTCAACCAGGAAACCAA |
| RPS_*rv* | GTGCAAGAGACCGGACATACC |
